# Prevalence and clinical consequences of Hepatitis E in patients who underwent liver transplantation for chronic Hepatitis C in the United States

**DOI:** 10.1186/s12879-015-1103-9

**Published:** 2015-09-02

**Authors:** Ludi Koning, Michael R. Charlton, Suzan D. Pas, Julie K. Heimbach, Albert DME Osterhaus, Kymberly D. Watt, Harry LA Janssen, Robert J. de Knegt, Annemiek A. van der Eijk

**Affiliations:** Department of Gastroenterology and Hepatology, Erasmus University Medical Center, Rotterdam, The Netherlands; Department of Viroscience, Erasmus University Medical Center, room NA-1019, Wytemaweg 80, 3015 CN Rotterdam, The Netherlands; Mayo Clinic Transplant Center, Rochester, MN USA; Intermountain Medical Center, Transplant Center, Salt Lake City, UT USA; Toronto Centre for Liver Disease, Toronto Western & General Hospital, University Health Network, Toronto, Canada

## Abstract

**Background:**

Infection with hepatitis E virus (HEV) in immunocompromised patients can lead to severe liver disease. Treatment options for HEV include peginterferon or ribavirin, routinely also used for the treatment of hepatitis C virus (HCV) infection.

We determined the prevalence and clinical consequences of HEV in United States (US) based patients who underwent liver transplantation (LT) for chronic HCV.

**Methods:**

Seroprevalence of HEV in 145 US LT recipients with a history of chronic HCV was determined pre-LT, 1, 3 and 5 years post-LT. All last available samples and all samples in IgM positive patients and post-LT IgG seroconverters were tested for HEV RNA.

**Results:**

Overall anti-HEV seroprevalence was 42 %. Five patients were HEV IgM positive pre-LT, one patient had IgM seroconversion post-LT and eight patients had IgG seroconversion post-LT. None of the tested samples were positive for HEV RNA. Eight out of nine of the post-LT seroconverters had been treated for HCV recurrence before or at the moment of seroconversion.

**Conclusions:**

LT recipients in the US are at risk of acquiring HEV. Post-LT HCV treatment with interferons and/or ribavirin may have protected patients against chronic HEV. With the arrival of new direct antiviral agents for the treatment of HCV and the elimination of peginterferon and ribavirin from HCV treatment regimens, the prevalence of chronic HEV in this population may rise again.

## Background

Hepatitis E virus (HEV), an enterically transmitted virus that is known for causing acute hepatitis, was first isolated in 1990 [1]. HEV is a non-enveloped virus with a single-stranded, positive sense RNA genome of approximately 7,500 base pairs and three partially overlapping open reading frames (ORF 1–3) [2]. Up to date, there are four genotypes prevalent known to infect humans. Genotype 1 and 2 are endemic in developing countries and mainly transmitted through contaminated water, while genotypes 3 and 4 are sporadically seen in industrialized countries and thought to be zoonotic of origin [3, 4].

While in most cases HEV infection is usually a self-limiting disease, in the past years multiple reports have emerged on immunocompromised patients, especially solid organ transplant (SOT) recipients, developing chronic HEV infection [5–7]. Reports on HEV infections in SOT recipients in the United States are scarce. Almost all well-defined cohort studies in industrialized countries on HEV infection in SOT recipients up to now have been done in Europe and these results cannot be automatically extrapolated to the situation in the US due to geographical and demographic differences. One study in HIV-infected liver and kidney transplant candidates in the US showed a pre-transplant seroprevalence of almost 20 % [8]. Estimates of the seroprevalence of HEV in the general population (including blood donors) in the US are reaching up to 22 % [9–13]. Swine-human contact is seen as an important possible source for HEV transmission in the US: HEV infection occurs in more than 80 % of some pig herds in the US [14] and HEV RNA has been found in pig feces on multiple US farms and pig livers sold in local US grocery stores [15–17]. Veterinarians working with swine are 1.5 times more likely to be HEV IgG positive than blood donors from the same area [10]. In US blood donors, an HEV seroprevalence of up to 22 % has been reported, making blood transfusions a likely mode of transmission as well [9–11].

Consequently, SOT recipients in the US are at risk of acquiring HEV infection. Early detection of HEV infection in these patients is crucial, since chronic HEV in SOT recipients can lead to rapid fibrosis and even cirrhosis [18–20].

Chronic HEV can be adequately managed by dose reduction of immunosuppressive medication or treatment with peginterferon alpha or ribavirin (RBV), compounds also used for the treatment of hepatitis C virus (HCV) infection [21–24]. Currently, no studies on HEV infection in HCV infected SOT recipients are available. On one hand, there is a risk of development of progressive or even fulminant liver disease when co-infection with HEV in these patients occurs. On the other hand, chronic HEV infection may be less prevalent due to treatment of HCV.

To gain more insight into the prevalence of hepatitis E infection in SOT recipients in the US and the influence of HCV treatment on the incidence and clinical course of HEV we conducted the current study in a cohort of patients with a history of HCV that underwent a liver transplantation in the Mayo Clinic in Rochester, Minnesota.

## Methods

### Patients and samples

From 1997 through 2010 serum samples were prospectively collected according to a standard protocol in patients with a history of chronic hepatitis C infection that underwent liver transplantation for end stage liver disease or hepatocellular carcinoma in the Mayo Clinic in Rochester, Minnesota (US). No donor organs were obtained from executed prisoners or other institutionalized persons. Samples were collected in 145 patients at four time points: pre-transplantation (baseline), 1, 3 and 5 years post-liver transplantation and subsequently stored at −70 °C. Each enrolled subject had consented in future testing of archived bio-samples. Samples were shipped to the Erasmus University Medical Center in Rotterdam (The Netherlands) for hepatitis E testing. The study protocol conforms to the ethical guidelines of the 1975 Declaration of Helsinki and was approved by the Institutional Review Board of the Mayo Clinic in Rochester, Minnesota (US).

### HEV specific antibody detection

First, all collected samples were tested for both HEV specific IgM and HEV specific IgG with the commercially available enzyme-linked immunosorbent assay (ELISA) (Wantai, Beijing, China), used according to the manufacturer’s instructions.

### HEV-RNA detection

Second, all samples in patients with HEV specific IgM antibodies and patients with HEV specific IgG seroconversion post-LT were tested for the presence of HEV RNA by an internally controlled quantitative real-time RT-PCR, described previously [6]. The RT-PCR has a lower limit of detection (95 % hit rate) of 143 IU/ml as determined by the 1st WHO standard for HEV RNA NAT-Based assays (6329/10, Paul Ehrlich Institute, Germany). Finally, all last available samples in the 145 patients were tested for HEV RNA to detect any ongoing active HEV infection at the last follow up point, since seroconversion in immunocompromised patients is often delayed and these patients might not have seroconverted yet.

## Results

**Fig. 1:**
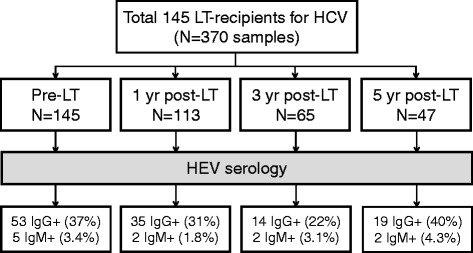
Sample distribution and Hepatitis E serology in 145 Liver Transplant Recipients. Abbreviations: LT, Liver Transplant; HEV, Hepatitis E Virus

A total of 370 samples in 145 LT-recipients were available for analysis. Distribution of samples over time and serology test results are given in Fig. 1. Baseline characteristics are given in Table 1.

**Table 1:** Baseline characteristics in LT recipients with a history of HCV infection

	HEV IgG positive *N* = 53	HEV IgG negative *N* = 92	p-value
Mean age, years	53 ± 7	51 ± 8	0.153
Male sex	45 (85 %)	64 (70 %)	*0.047*
Caucasian ethnicity	47 (88 %)	79 (84 %)	0.615
Residing in Minnesota	29 (53 %)	35 (37 %)	0.077
History of alcohol abuse	26 (49 %)	34 (37 %)	0.166
HCV genotype 1	28 (67 %)	63 (83 %)	0.066

*LT* liver transplant, *HCV* Hepatitis C virus, *HEV* Hepatitis E virus

### IgG seroconversion

A total of 61 patients (42 %) had HEV specific IgG antibodies at some point from baseline up to last follow up. Seroprevalence of HEV at baseline was higher in men compared to women, however, overall seroprevalence from baseline up to last follow up did not differ between sexes: *N* = 60 (40 %) for men and *N* = 18 (33 %) for women (*p* = 0.418). Of the 53 patients that had HEV specific IgG antibodies at baseline, 46 had at least one follow-up sample post-LT. Fifteen of these patients (33 %) with IgG antibodies at baseline had IgG loss at some point post-LT. A total of 125 patients had a baseline sample and at least one follow up sample available. Eight patients (6.4 %) were IgG negative at baseline and showed IgG seroconversion post-LT; four at year 1, one at year 3 and three at year 5 post-LT. Two of these eight patients had IgG loss after post-LT seroconversion.

### IgM seroconversion

Only six out of 145 patients (4.1 %) had IgM antibodies against HEV. Five patients had IgG and IgM antibodies against HEV at baseline and one patient, who had been IgG positive from baseline up to 5 years post-LT, seroconverted IgM at year 5 post-LT (see also Table 2). One of the 6 IgM-positive patients had IgG and IgM loss post-LT, another patient experienced IgG loss but remained IgM positive.

**Table 2:** Characteristics of nine post-LT HEV seroconverting patients with a history of HCV infection

	Age at LT	Gender	Ethnicity	LT year	Type of serologic conversion	Conversion year (from LT)	Post-LT HCV treatment	Treatment year post-LT	Treatment medication
Pt 1	46	Male	Caucasian	1998	IgM	5	Yes	1-5	IFN + RBV
Pt 2	48	Male	Caucasian	1998	IgG	1	Yes	0-1	IFN + RBV
Pt 3	39	Male	Native Hawaiian	2000	IgG	5	Yes	2-3	IFN + RBV
Pt 4	36	Female	Caucasian	2001	IgG	1	Yes	0-1	IFN + RBV
Pt 5	47	Male	Other NS	2001	IgG	1	Yes	0	IFN
Pt 6	51	Male	Caucasian	2001	IgG	5	Yes	2-4	IFN + RBV
Pt 7	47	Male	Caucasian	2003	IgG	3	Yes	1-4	IFN + RBV
Pt 8	51	Male	Caucasian	2004	IgG	5	Yes	0-3	IFN + RBV
Pt 9	54	Male	Caucasian	2009	IgG	1	No	N/A	N/A

*LT* Liver transplant, *HEV* Hepatitis E virus, *HCV* Hepatitis C virus, *IFN* (peg) interferon, *RBV* ribavirin, *Other NS* Other than Caucasian, Hispanic, Black or Asian, not specified, *N/A* not applicable

### Post-LT HEV infection

None of the last available samples in the 145 patients or any of the samples in the IgM positive and IgG seroconverters were positive for HEV RNA. We hypothesized that HCV infected LT-recipients were protected against chronic HEV infection due to concurrent treatment with either (peg) interferon or RBV for the treatment of HCV. To test this hypothesis we selected the patients that were most likely to have had active infection post-LT, namely: the patients that had IgM and/or IgG seroconversion post-LT. All 8 IgG seroconverters and the single IgM positive patient that seroconverted after LT met this definition and were further explored (see Table 2).

All but one patient was treated with (peg) interferon and/or RBV before or at the time of seroconversion.

## Discussion

In this study we examined the prevalence and impact of serological evidence of HEV infection among a well-defined cohort of US liver transplant recipients with a history of HCV infection. The impact of HEV infection among solid organ transplant recipients is likely to be greatest among patients with pre-existing liver injury such as caused by chronic HCV, since acute on chronic infection will most likely lead to an aggravated clinical course. Post-transplant HCV infection is the most common cause of graft loss among liver transplant recipients, raising the possibility that the consequences of post-transplant HEV infection might be most readily apparent in this group of patients. Our analysis of HEV infection in a relatively large cohort of liver transplant recipients with HCV infection has produced several potentially important results that merit detailed consideration. The first important observation is that in this cohort, more than four out of ten liver transplant recipients with a history of HCV infection have been in contact with HEV. This finding highlights that these patients are at considerable risk of exposure to HEV, which may lead to chronic infection or accelerated liver disease. Indeed, we showed that transplant recipients in the US are at risk of acquiring HEV infection following liver transplantation, considering that nine out of 125 patients (7.2 %) with at least one follow up sample had HEV seroconversion post-LT.

There are several explanations why over four out of ten patients in this cohort of US liver transplant recipients with a history of HCV infection have HEV antibodies. Since HCV is a blood borne disease, HEV infection may share the same transmission route as HCV. An association between intravenous drug use in HCV infected patients and the prevalence of HEV IgG antibodies has been inconsistently reported [25, 26]. Another possible transmission route is through transfusion with blood products. There is a considerable seroprevalence of HEV in blood donors in the US with anti-HEV antibodies of up to 22 %, though no active infection in US cohorts has been found [9–11]. In contrast, in European cohorts, HEV RNA was found in plasma and blood donors [27–33] leading to HEV infection in patients receiving those blood components [33]. As long as blood products are not routinely screened for HEV RNA, US physicians should be aware of this possible transmission route. LT recipients usually have received multiple blood products over the course of their disease. HCV treated LT recipients are even more likely of receiving blood transfusion due to treatment-induced anemia, further increasing the chance of receiving blood products from an HEV viremic donor. Patients are also at risk of acquiring HEV through infection of the donor organ as a recent report on a liver transplant recipient showed [34]. Finally, the average age in this cohort was 51.8 years and several studies have shown that HEV seroprevalence increases with age [10, 11, 35, 36].

One of the unexpected observations in our analysis was that, despite a substantial number of patients seroconverting to anti-HEV IgM and/or anti-HEV IgG following liver transplantation, we did not find any active HEV infection in this cohort. It is possible that HEV viremia was missed in our study due to the time between serum draws that were available for analysis. HEV seroconversion may have occurred with (spontaneous) clearance of active HEV infection and subsequent loss of HEV antibodies over time due to immunosuppressive therapy, as illustrated by HEV IgG loss post-LT in one third of patients with HEV IgG antibodies at baseline. We may also have missed patients that did not seroconvert at all despite infection due to their immunosuppressive state, since HEV seroconversion is often delayed or even absent in these patients [6, 24, 37, 38]. Patients that obtained HEV infection after the last follow up sample and subsequently died will have been missed as well. Our results may, therefore, have underestimated the severity of acute HEV infection.

We used the most sensitive HEV serologic test available at the moment [39–41] and an internally controlled quantitative real-time RT-PCR, which has been an effective and reproducible tool for detecting HEV RNA in other cohorts [35, 42]. The effects of immunosuppression, which include suppression of antibody production and an increase of levels of viremia (e.g. for HCV, HBV, CMV), may have resulted in an underestimation of the seroprevalence of anti-HEV in this cohort but should not have affected the numbers regarding prevalence of active HEV infection. Our results should generally be viewed as reassuring in that no active HEV infection was found in any of the patients at last follow up. Moreover, HEV infection in SOT recipients does not always progress to chronicity [37]. A further consideration in the lack of active HEV infection in our analysis is that eight out of nine post-LT anti-HEV seroconverters were treated with either RBV or (peg) interferon. Both of these agents are known to be effective therapies for HEV infection and may have contributed to HEV clearance. As HCV treatment initiation is often based on clinical parameters indicating (recurrent) hepatitis, it is possible that superinfection with HEV may have contributed to the likelihood of initiation of anti-HCV therapy. Soon, new antiviral agents, including interferon- and ribavirin-free protocols, against HCV will become available for the treatment of HCV infection. The disappearance of interferon and RBV from HCV treatment regimens may increase the risk of acquiring chronic HEV and subsequent development of fibrosis and cirrhosis, as these new agents will most likely not have activity against HEV. HCV liver transplant recipients not treated for HEV are currently still at risk of developing chronic HEV.

Limitations of this study include a lack of a comparative non-HCV infected group of liver transplant recipients, the retrospective nature of the study and patients that were lost to follow up due to their transferring care to a local hospital.

## Conclusions

LT recipients in the US are at risk of acquiring HEV. Therefore, screening of HEV in LT recipients should be carried out routinely, especially when there are clinical signs of (progressive) hepatitis. Evaluation of immunocompromised patients should include HEV RNA testing, since antibody detection is often delayed in these patients. Due to the lack of FDA-approved HEV RNA tests and high interlaboratory variability in PCR performance [43], we recommend to have samples tested in a laboratory with extensive experience and up to date assays. HCV infected LT recipients may be protected against the development of chronic HEV through treatment against HCV. However, HEV infection should be best managed through dose reduction of immunosuppressive medication and/or treatment with low-dose RBV [44], avoiding overtreatment of HCV infected patients. Due to the arrival of new interferon- and RBV-free HCV regimens not active against HEV, HCV infected LT recipients will again be at risk of acquiring (chronic) HEV in the nearby future.

## Abbreviations

HEV: Hepatitis E virus
RBV: Ribavirin
HCV: Hepatitis C virus
US: United States
LT: Liver transplant
ORF: Open reading frames
SOT: Solid organ transplant
ELISA: Enzyme-linked immunosorbent assay
WHO: World Health Organization
